# Hybrid Feature Mammogram Analysis: Detecting and Localizing Microcalcifications Combining Gabor, Prewitt, GLCM Features, and Top Hat Filtering Enhanced with CNN Architecture

**DOI:** 10.3390/diagnostics14151691

**Published:** 2024-08-05

**Authors:** Miguel Alejandro Hernández-Vázquez, Yazmín Mariela Hernández-Rodríguez, Fausto David Cortes-Rojas, Rafael Bayareh-Mancilla, Oscar Eduardo Cigarroa-Mayorga

**Affiliations:** 1 Departamento de Tecnologías Avanzadas, UPIITA-Instituto Politécnico Nacional, Av. Instituto Politécnico Nacional 2580, Ciudad de México 07340, Mexicoyazmin.hernandez@cinvestav.mx (Y.M.H.-R.); 2 Departamento de Ingeniería Eléctrica/Sección de Bioelectrónica, Centro de Investigación y de Estudios Avanzados del IPN, Av. Instituto Politécnico Nacional 2508, Col. San Pedro Zacatenco, Gustavo A. Madero, Ciudad de México 07360, Mexico; fdavid.cortezr@cinvestav.mx

**Keywords:** microcalcification detection, Convolutional Neural Networks (CNNs), hybrid feature extraction

## Abstract

Breast cancer is a prevalent malignancy characterized by the uncontrolled growth of glandular epithelial cells, which can metastasize through the blood and lymphatic systems. Microcalcifications, small calcium deposits within breast tissue, are critical markers for early detection of breast cancer, especially in non-palpable carcinomas. These microcalcifications, appearing as small white spots on mammograms, are challenging to identify due to potential confusion with other tissues. This study hypothesizes that a hybrid feature extraction approach combined with Convolutional Neural Networks (CNNs) can significantly enhance the detection and localization of microcalcifications in mammograms. The proposed algorithm employs Gabor, Prewitt, and Gray Level Co-occurrence Matrix (GLCM) kernels for feature extraction. These features are input to a CNN architecture designed with maxpooling layers, Rectified Linear Unit (ReLU) activation functions, and a sigmoid response for binary classification. Additionally, the Top Hat filter is used for precise localization of microcalcifications. The preprocessing stage includes enhancing contrast using the Volume of Interest Look-Up Table (VOI LUT) technique and segmenting regions of interest. The CNN architecture comprises three convolutional layers, three ReLU layers, and three maxpooling layers. The training was conducted using a balanced dataset of digital mammograms, with the Adam optimizer and binary cross-entropy loss function. Our method achieved an accuracy of 89.56%, a sensitivity of 82.14%, and a specificity of 91.47%, outperforming related works, which typically report accuracies around 85–87% and sensitivities between 76 and 81%. These results underscore the potential of combining traditional feature extraction techniques with deep learning models to improve the detection and localization of microcalcifications. This system may serve as an auxiliary tool for radiologists, enhancing early detection capabilities and potentially reducing diagnostic errors in mass screening programs.

## 1. Introduction

Breast cancer is the most common disease among women worldwide, with incidences exceeding 2 million new cases and approximately 600,000 deaths per year [1]. This condition shows significant variations in its incidence rate depending on the region, with higher figures in developed nations. Specifically, in Mexico, breast cancer represents the leading cause of cancer death among women, with about 28,000 new diagnoses and 6000 deaths annually [2,3]. Recently, there has been a drive to improve early diagnosis of breast cancer through improved access to screening programs. Implementing national screening programs and adopting technologies such as digital mammography has helped increase detection efficiency and improve treatment outcomes [4]. Early detection is vital for treatment success and for raising survival rates. Screening programs, including mammography and clinical breast examinations, are effective in decreasing the mortality associated with this cancer. However, in many low- and middle-income countries, significant challenges persist in terms of access to both screening and treatment due to disparities in the availability of health and oncology services [5].

One of the early symptoms of breast cancer is the presence of microcalcifications (MCs). MCs are calcium deposits inside the breast tissue that are not palpable but can be visualized by mammography. They may occur in clusters and be heterogeneous. They are mostly present in postmenopausal women and are usually associated with tissue necrosis and cellular residue. Most microcalcifications are caused by benign pathologies, but some others are caused by malignant pathologies, such as non-palpable breast cancer, where 55% of cases present visible microcalcifications. This percentage expands with ductal carcinoma in situ by 85–95%, where microcalcifications are observed in branched, linear, or fine forms. This condition is considered an intraductal, early-stage, localized cancer that does not cross the basement membrane and does not yet metastasize. If left untreated, it can progress to invasive ductal carcinoma, being the most common type of cancer and accounting for 75% of all cases during the 10 to 20 years after initial diagnosis [6,7].

Given the statistics previously presented, it is evident that the detection of microcalcifications, particularly in dense breast tissue, represents a significant challenge in breast cancer screening. Dense breast tissue, which can obscure microcalcifications on mammograms, is a well-known factor that reduces the sensitivity of mammographic imaging. Studies have shown that dense breast tissue is present in approximately 40–50% of women undergoing mammography, and this density significantly complicates the identification of small calcifications indicative of early breast cancer [8,9]. Moreover, it is estimated that a substantial percentage of mammograms—up to 20–30%—are not accurately diagnosed with the presence of microcalcifications due to multifactorial issues. Key factors contributing to this diagnostic gap include radiologist fatigue, insufficient medical personnel, and the prevalence of false-negative results. Radiologist fatigue, especially in high-volume screening environments, leads to decreased accuracy in reading mammograms, increasing the likelihood of missed diagnoses [10,11,12]. Additionally, the variability in imaging equipment and settings further complicates the detection process, as these factors can affect image quality and consistency. This issue is further combined by the shortage of trained radiologists, which places additional strain on existing personnel and heightens the risk of diagnostic errors. False-negative mammogram results, where microcalcifications are present but not identified, pose a critical challenge. These errors occur for several reasons, including the subtle appearance of microcalcifications and their similarity to benign conditions, which can mislead even experienced radiologists. Studies have highlighted that false-negative rates can be as high as 15–30%, underscoring the need for advanced diagnostic tools and methods to support radiologists in accurately identifying these critical markers of early breast cancer [13].

Several efforts have been made on different fronts, particularly in medical imaging, with mammography detection being the gold standard. However, due to the previously mentioned challenges, Artificial Intelligence (AI) could contribute to improving detection and, as a long-term goal, diagnosis. The idea of contributions from AI is to provide an auxiliary first-line tool so that radiologist specialists have a starting point for the diagnosis of each patient [14,15,16,17]. Efforts have been made in this field over the past decades, mainly with the implementation of machine learning (ML) and deep learning (DL) algorithms.

However, the implementation of AI is not a trivial task. Pathology detection based on medical imaging requires intermediate processes and extensive expertise in several methods and techniques. For instance, AI-based detection systems require a large database with an adequate number of samples to generalize the problem. Each algorithm may require different quantities depending on specific objectives. Ideally, the dataset should be balanced between the classes to be classified or detected. Class imbalance could lead to significant errors in classification performance. Also, normalizing the image database is a relevant stage, commonly referred to as the preprocessing stage, ensuring that all input images to the system have consistent spatial conditions, appropriate contrast, and are without interferences. This scenario is particularly relevant when multiple sources are used to compile a unified database, as different mammography equipment can produce images with different characteristics. Subsequently, the architecture of the classification stage must be designed according to the specific applications or problems to generalize the detection or classification task effectively. While deep learning, specifically Convolutional Neural Networks (CNNs), can perform well, they remain a black box, and the mathematical models and generalization processes are not always comprehensible [18]. This factor has contributed to skepticism in the medical community regarding the implementation of Computer-Aided Diagnosis (CAD) systems, despite promising studies with high detection performance [19]. In recent years, the trend has been towards the implementation of Explainable Artificial Intelligence (XAI), which aims to analyze medical images from a perspective that aligns more closely with human reasoning, based on descriptive features in morphological and structural characteristics [20,21,22,23,24,25,26,27].

In this context, this work presents a method for the detection and localization of MCs based on mammogram images, addressing class imbalance through data augmentation techniques such as scaling and rotation transformations. Additionally, this work introduces an approach for detection using descriptive features based on the identification of regions that stand out as bright spots, using hybrid feature filters: Prewitt, Gabor, and Gray Level Co-occurrence Matrix (GLCM). Prewitt, Gabor, and Gray Level Co-occurrence Matrix (GLCM) filters were chosen for feature extraction due to their distinct advantages in detecting and analyzing microcalcifications in mammograms. The Prewitt filter is effective in edge detection, highlighting regions with high spatial gradients. The Gabor filter captures texture information and features with specific orientations and frequencies. The GLCM method analyzes the spatial relationship of pixel intensities, highlighting areas with varying densities and patterns. Compared to other filters, these three provide a balanced combination of edge detection, texture analysis, and statistical feature extraction, making them well-suited for the challenges of MC analysis. These descriptive features of the mammograms were then used as input data for a CNN with intermediate maxpooling stages to identify pixels indicative of calcification, and activation functions including Rectifier Linear Unit (ReLU) and sigmoid. Key points of our findings include:

- Data Augmentation: Implemented scaling and rotation transformations to mitigate class imbalance, ensuring a more robust training dataset.
- Unified Database: Created a unified database from multiple sources to tackle class imbalance, providing a more comprehensive dataset for training and testing.
- Feature Extraction: Utilized Prewitt, Gabor, and GLCM filters to highlight potential microcalcification areas, enhancing the visibility of these regions.
- CNN Architecture: Designed a CNN with intermediate maxpooling layers to effectively process and analyze the extracted features.
- Activation Functions: Employed ReLU and sigmoid activation functions to improve the detection accuracy of microcalcifications.
- Performance Metrics: Achieved a sensitivity of 0.8214 and specificity of 0.9147, demonstrating the effectiveness of the proposed method in detecting and localizing microcalcifications in mammograms.

## 2. Literature Review

Numerous CAD systems have been developed by researchers to enhance the early detection of breast cancer through mammogram images. This manuscript aims to present a comprehensive overview of existing methodologies that leverage machine learning and deep learning algorithms for MC detection using deep CNNs. In recent years, the application of deep learning networks has significantly advanced the classification of digital medical images, facilitating object detection and disease prediction. The detection and classification of MCs in mammograms is a critical task in the early diagnosis of breast cancer. Various CAD systems have been developed to enhance the accuracy and efficiency of this process. In recent years, the application of deep learning (DL) and machine learning (ML) techniques has significantly advanced the field, enabling more precise and automated detection methods. Numerous studies have explored different approaches to improve the detection and classification of MCs.

Luna-Lozoya et al. (2024) utilized the INbreast database, comprising 410 mammograms, and applied data augmentation techniques. Their method involved a lightweight CNN with two convolutional layers, a global pooling layer, and a sigmoid function for binary classification, achieving an impressive accuracy of 99.30% and sensitivity of 95.00% [28]. Qing Lin (2023) worked with a private clinical database of 720 images, employing DenseNet with transfer learning. This approach resulted in an accuracy of 92.30% and a sensitivity of 88.70% [29]. Yurdusev et al. (2023) used the DDSM database with 500 images, applying a difference filter and Yolov4 deep learning model. Their study reported an accuracy of 97.67% and a sensitivity of 98.36% [30]. Singh et al. (2022) used a private clinical dataset of 1150 mammograms and implemented transfer learning with ResNet-50, achieving an accuracy of 89.50% and a sensitivity of 85.20% [31]. Xiao (2021) utilized data from the Department of Radiology at Nanjing Medical University Affiliated Hospital, working with 462 DBT volumes from 236 patients. They combined 2D ResNet34 and 3D ResNet in an ensemble CNN, achieving an accuracy of 82.00% and a sensitivity of 84.38% [32]. Rehman et al. (2021) worked with 960 images from the DDSM database (480 benign and 480 malignant), applying a CNN with transfer learning and data augmentation, achieving an accuracy of 90.00% and a sensitivity of 87.50% [33]. Castro Tapia et al. (2021) also used the DDSM database, focusing on 4189 regions of interest (ROIs). They combined traditional feature extraction (Gabor, Prewitt, GLCM) with a CNN, achieving an accuracy of 85.40% and a sensitivity of 83.70% [34]. Chen et al. (2020) used 2200 images from Chung Shan Medical University Hospital, with a Cascade Adaboost and CNN approach, reporting an accuracy of 85.00% and a sensitivity of 82.00% [35]. Vancheri et al. (2019) utilized the MIAS database with 283 mammograms, employing two CNNs for the detection and segmentation of regions of interest, achieving an accuracy of 98.22% and a sensitivity of 97.47% [36]. Hekim et al. (2019) used various hospital datasets with 219 mammograms and implemented a pixel assignment-based spatial filter, achieving an accuracy of 90.90% and a sensitivity of 88.40% [37].

While these studies have significantly advanced the field of MC detection, our approach introduces a contribution that distinguishes it from the existing literature. We propose a hybrid method that integrates traditional feature extraction techniques (Gabor, Prewitt, GLCM) with advanced CNN architectures. This combination influences the strengths of both traditional and DL methods. Our study addresses class imbalance by creating a unified database from multiple sources, ensuring a more comprehensive and balanced dataset for training and testing. We employ data augmentation techniques, including scaling and rotation transformations, to further enhance the robustness and generalization capabilities of our model. By addressing the limitations of previous studies and introducing these innovations, our work aims to contribute significantly to the field of breast cancer diagnosis, providing radiologists with more reliable and interpretable tools for early detection. Table 1 summarizes several studies on the detection and classification of microcalcifications in mammograms, detailing the authors, the database origin, the number of mammograms used, whether augmentation techniques were applied, the architecture and methods employed, as well as the reported accuracy and sensitivity of each method.

## 3. Materials and Methods

**Figure 1:**
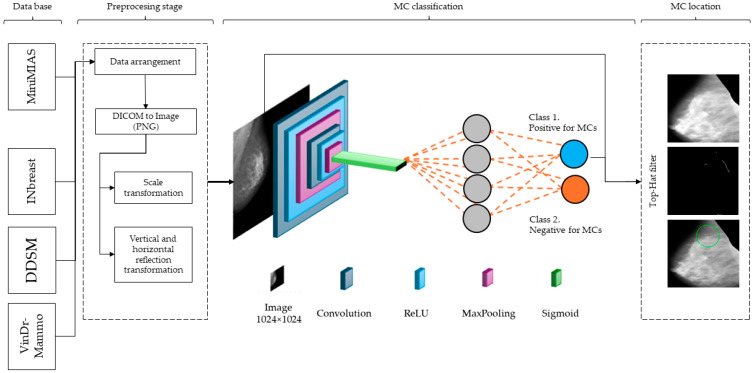
Overview of the CNN architecture summarizing the methods section. It illustrates the process from input mammogram, through convolutional, ReLU, and maxpooling layers, to the final classification of mammograms as positive or negative for MCs. The green circle marks the location of the MCs.

This section describes the nature of the database used for training, validation, and testing, as well as a detailed description of the methodology at each stage to test the hypothesis that descriptive features based on intensity characteristics, enhanced with a CNN, could achieve acceptable performance for the detection and localization of microcalcifications (MCs). Figure 1 presents an overview of the process.

### 3.1. Dataset Characteristics, Augmentation, and Preprocessing Stage

In this section, we provide a detailed overview of the mammographic images and their characteristics used in our study. The dataset comprises mammograms sourced from multiple medical institutions, ensuring a diverse and comprehensive collection of images. Our dataset includes both benign and malignant cases, with a variety of microcalcification appearances to support robust model training and evaluation. The dataset integrates mammograms from several publicly available databases, including INbreast, VinDr-Mammo, MIAS, and DDSM, totaling 9098 mammograms. The INbreast database contributed 410 mammograms [38], VinDr-Mammo provided 5000 mammograms [39], MIAS included 322 mammograms [40], and DDSM contributed 3366 mammograms [41]. Each mammogram in the dataset was carefully annotated by experienced radiologists to provide accurate ground truth labels for microcalcifications. The annotations include detailed information about the location, size, and type of microcalcifications, ensuring high-quality and reliable data for training and testing. Table 2 provides an outlook of the database considered for this paper.

As can be seen in Table 2, the number of cases identified as positive for microcalcification represents approximately one tenth (16.16%) of the total universe. To address the issue of class imbalance, we created a unified and balanced dataset by combining images from different sources and applying data augmentation techniques. Specifically, we used different scaling factors (i.e., 0.8, 1.0, and 1.2) to simulate sizes of MCs, ensuring the model could detect MCs of different magnitudes. Mathematically, the scaling transformation is described by Equation (1).

(1) $$I^{\prime }\left(x,\;y\right)=I\left(s_{x}\cdot x,\;s_{y}\cdot y\right)$$

where *I* is the original image and *I’* is the scaled image, $s_{x}$ and $s_{y}$ are the scaling factors in the *x* and *y* dimensions, respectively. Afterwards, the images were rotated at angles of 90°, 180°, and 270° to improve the recognition of MCs regardless of their orientation. The rotation transformation is given by Equation (2).

(2) $$\;I^{\prime }\left(x,\;y\right)=I\left(x\;cos\;\theta -y\;sin\;\theta ,\;x\;sin\;\theta +y\;cos\;\theta \right)$$

where *θ* is the rotation angle. Lastly, reflection transformations include both vertical and horizontal reflections, expressed by Equations (3) and (4).

(3) $$I^{\prime }\left(x,y\right)\;=\;I\left(x,\;H\;-\;y\right)\;$$

(4) $$I^{\prime }\left(x,y\right)=I\left(W-x,y\right)$$

where *H* and *W* are the height and width of the image, respectively. These transformations doubled the dataset by creating mirror images of the original data, thereby helping the model generalize better by learning from a wider variety of patterns. This approach ensures that the dataset is representative of real-world scenarios and enhances the generalization capabilities of our model. After applying data augmentation, a total of 2748 mammography positive for MCs were obtained. The classes were balanced with an approximately equal number of negative cases, resulting in 2942 mammography. This decision was based on the necessity to address class imbalance, which can lead to biased model predictions. By ensuring a balanced dataset, the model can learn the distinguishing features of both classes more effectively, improving its generalization capabilities and providing reliable performance metrics. The balanced dataset brought the total to 5690 mammography, which was then divided into training, validation, and test sets with 70% for training, 20% for validation, and 10% for testing, as detailed in Table 3. This balanced approach ensures that the model is trained effectively, validated thoroughly, and tested reliably, providing robust performance metrics and enhancing its generalizability to unseen data.

After the data augmentation stage, a preprocessing stage is necessary to homogenize the input data for the classification stage. This preprocessing step is crucial for ensuring that all mammographic images have uniform characteristics, thereby improving the consistency and accuracy of the classification model. These preprocessing steps are essential to prepare the mammographic images for effective classification. Data homogenization not only improves the model’s accuracy but also reduces the risk of overfitting and enhances the model’s ability to generalize when working with data from different sources.

The homogenization of data includes several important steps.

- Grayscale Conversion: All images are converted to grayscale to perform feature extraction processing. Although the images may appear to be grayscale to the naked eye, they are not; this is known as false RGB.
- Image Resizing: Images are resized to a 1024 × 1024 resolution to ensure that the model receives consistent inputs, regardless of the original size variations of the mammograms. The resolution was chosen based on a study where we applied Convolutional Neural Networks (CNNs). This resolution meets the requirements for an adequate number of features in the first layer of the CNN, ensuring sufficient detail is preserved for detecting MCs while maintaining computational efficiency.
- Contrast Normalization: Contrast enhancement techniques, such as the Value of Interest Look-Up Table (VOI-LUT), are applied to highlight regions of interest and improve the visibility of microcalcifications.
- Artifact Removal: This step was based on a 4-connected island detector, isolating only the largest region corresponding to the breast, and assigning the same background value (black) to the rest of the islands. This way, artifacts such as medical annotations or those from the equipment itself were eliminated.

To preprocess the images, we converted them to grayscale using the MATLAB R2023b function “*rgb2gray*”. This function calculates a weighted sum of the red, green, and blue channels according to Equation (5).

(5) $$I_{gray}=0.2989\cdot I_{R}+0.5870\cdot I_{G}+0.1140\cdot I_{B}$$

where $I_{gray}$ is the resulting grayscale image, and $I_{R}$, $I_{G}$, and $I_{B}$ are the red, green, and blue channels of the original RGB image. This conversion simplifies the data by reducing it to a single intensity value per pixel, focusing on intensity variations. By using grayscale images, we ensured that edge detection, texture analysis, and other feature extraction methods leading to detect MCs cases.

Artifact removal was performed using a 4-connected island detector to enhance the quality of the mammogram images. The process involved the following steps:

Island detection: the algorithm scans the image to identify all contiguous regions (islands) of pixels.

Largest island identification: the largest island, which corresponds to the breast tissue, is identified.

Artifact removal: smaller islands, representing artifacts such as mammographic annotations, labels, and equipment marks, are removed by setting their pixel values to the background value (black).

This method effectively eliminates unwanted artifacts, ensuring that only the relevant breast tissue is retained for subsequent feature extraction and analysis.

### 3.2. Feature Extraction Kernels for Microcalcification Detection in Mammograms

This section discusses the methods for detecting positive cases of MCs and their subsequent localization. This stage was based on implementing the extraction of descriptive features to detect microcalcifications by size, which vary between 0.1 and 1 mm in diameter. Additionally, their shape and morphology could influence their significance in terms of irregularity. Some suspicious calcifications might have greater density than the surrounding tissues, with scattered, clustered, segmental, linear, or regional. Changes in the tissue, such as distortion or the presence of a mass, can be significant indicators when evaluating microcalcifications.

In this regard, three types of filters were proposed to mimic the detection of the characteristics: Prewitt, Gabor, and GLCM. These filters have the particularity of enhancing the visibility of microcalcifications by emphasizing different aspects of the mammogram. The Prewitt filter is used for edge detection and helps highlight the boundaries of microcalcifications by identifying areas with high spatial gradients. It is effective in enhancing linear and irregular patterns, making it easier to detect microcalcifications that may have indistinct edges [42]. The Gabor filter, known for its ability to capture texture information, can enhance the visualization of microcalcifications with specific orientations and frequencies. It is particularly useful for detecting microcalcifications that have distinct texture patterns, improving their contrast against the background tissue [43]. The Gray Level Co-occurrence Matrix (GLCM) method focuses on the textural features of the mammogram by analyzing the spatial relationship of pixel intensities. GLCM can highlight areas with varying densities and patterns, which are indicative of suspicious microcalcifications. It provides detailed information on the texture and structure of the tissue, aiding in the differentiation of benign and malignant calcifications [44]. By combining these filters, the algorithm can effectively enhance the relevant density features associated with microcalcifications, aiding their detection and localization.

The Prewitt kernel was employed as the first convolution element. This kernel is an adaptation of the discrete Laplacian matrix and is specifically used to identify pixels exhibiting abrupt intensity changes in the image. Its relevance lies in the fact that microcalcifications often appear as white spots in mammograms. Equations (6) and (7) present the horizontal and vertical kernels.

(6) $${Prewitt}_{x}=\left[\begin{array}{ccc} -1 & 0 & 1\\ -1 & 0 & 1\\ -1 & 0 & 1 \end{array}\right]$$

(7) $${Prewitt}_{y}=\left[\begin{array}{ccc} -1 & -1 & -1\\ 0 & 0 & 0\\ 1 & 1 & 1 \end{array}\right]$$

For the next kernel, we used the Gabor filter, which describes properties in terms of spatial and frequency domains. Five filters were utilized with orientations ranging from 0° to 180°, in 45° increments, to cover various positions. The Gabor filter is employed to capture spatial and frequency domain properties of microcalcifications in mammograms. This filter is effective in detecting edges and textures, which are crucial for identifying microcalcifications. The Gabor filter is defined by a sinusoidal wave modulated by a Gaussian envelope, characterized by its orientation, frequency, and phase. The Gabor filter is expressed in Equation (8).

(8) $$G\left(x,\;y;\;\lambda ,\;\theta ,\;\psi ,\;\sigma ,\;\Upsilon \right)=\exp\left(-\frac{{x^{\prime }}^{2}+\Upsilon^{2}{y^{\prime }}^{2}}{{2\sigma }^{2}}\right)\cos\left(2\pi \frac{x^{\prime }}{\lambda }+\psi \right)$$

where *x’* is defined as $xcos\left(\theta \right)+ysin\left(\theta \right)$, and *y’* is defined as $-xsin\left(\theta \right)+ycos\left(\theta \right)$, λ is the wavelength (frequency) of the sinusoidal factor, *θ* is the orientation of the normal to the parallel stripes of the Gabor function, ψ is the phase offset, *σ* is the standard deviation of the Gaussian envelope, and γ is the spatial aspect ratio, specifying the ellipticity of the support of the Gabor function. A frequency of 0.625 was set, considering the typical size of a microcalcification, which can measure approximately 1 mm. The Gabor filter matrices for different orientations are generated by applying *x*’ and *y’*. Each matrix corresponds to a specific orientation *θ*. For instance, matrices for orientations of 0°, 45°, 90°, 135°, and 180° are generated by setting, as presented in Equations (9)–(13).

(9) $$\mathrm{Gabor}\;\mathrm{filter}\;\mathrm{for}\;\theta =0,\left[\begin{array}{ccccc} 0.1105 & 0.2368 & 0.2921 & 0.2368 & 0.1105\\ 0.2368 & 0.5101 & 0.6291 & 0.5101 & 0.2368\\ 0.2921 & 0.6291 & 0.7726 & 0.6291 & 0.2921\\ 0.2368 & 0.5101 & 0.6291 & 0.5101 & 0.2368\\ 0.1105 & 0.2368 & 0.2921 & 0.2368 & 0.1105 \end{array}\right]$$

(10) $$\mathrm{Gabor}\;\mathrm{filter}\;\mathrm{for}\;\theta =\frac{\pi }{4},\;\left[\begin{array}{ccccc} 0.0000 & 0.0000 & 0.1105 & 0.2368 & 0.1105\\ 0.0000 & 0.0000 & 0.2368 & 0.5101 & 0.2368\\ 0.1105 & 0.2368 & 0.5101 & 0.6291 & 0.2921\\ 0.2368 & 0.5101 & 0.6291 & 0.5101 & 0.2368\\ 0.1105 & 0.2368 & 0.2921 & 0.2368 & 0.1105 \end{array}\right]$$

(11) $$\mathrm{Gabor}\;\mathrm{filter}\;\mathrm{for}\;\theta =\frac{\pi }{2},\;\left[\begin{array}{ccccc} -0.1105 & -0.2368 & 0.0000 & 0.2368 & 0.1105\\ -0.2368 & -0.5101 & 0.0000 & 0.5101 & 0.2368\\ 0.0000 & 0.0000 & 0.0000 & 0.0000 & 0.0000\\ 0.2368 & 0.5101 & 0.0000 & 0.5101 & 0.2368\\ 0.1105 & 0.2368 & 0.0000 & 0.2368 & 0.1105 \end{array}\right]$$

(12) $$\mathrm{Gabor}\;\mathrm{filter}\;\mathrm{for}\;\theta =\frac{3\pi }{4},\;\left[\begin{array}{ccccc} 0.1105 & 0.2368 & 0.2921 & 0.2368 & 0.1105\\ 0.2368 & 0.5101 & 0.6291 & 0.5101 & 0.2368\\ 0.0000 & 0.0000 & 0.0000 & 0.0000 & 0.0000\\ -0.2368 & -0.5101 & 0.0000 & 0.5101 & 0.2368\\ -0.1105 & -0.2368 & 0.0000 & 0.2368 & 0.1105 \end{array}\right]$$

(13) $$\mathrm{Gabor}\;\mathrm{filter}\;\mathrm{for}\;\theta =\pi ,\;\left[\begin{array}{ccccc} 0.1105 & 0.2368 & 0.2921 & 0.2368 & 0.1105\\ 0.2368 & 0.5101 & 0.6291 & 0.5101 & 0.2368\\ 0.1105 & 0.2368 & 0.2921 & 0.2368 & 0.1105\\ 0.0000 & 0.0000 & 0.0000 & 0.0000 & 0.0000\\ -0.1105 & -0.2368 & 0.0000 & 0.2368 & 0.1105 \end{array}\right]$$

The preprocessed image is then convolved with the Gabor filter matrices. Convolution is a fundamental mathematical operation in image processing, where the filter matrix (kernel) is systematically moved across the entire image. At each position, the dot product of the kernel and the overlapping region of the image is computed. This involves multiplying each element of the kernel by the corresponding pixel value in the image and then summing the results to produce a single output pixel. The result is a new image (feature map) that emphasizes certain features based on the characteristics of the kernel. For detecting microcalcifications, this operation is performed using Gabor filters, which are particularly effective for capturing spatial and frequency information. Each orientation of the Gabor filter is designed to respond maximally to edges and textures in the corresponding direction.

- A 0° orientation detects vertical features.
- A 45° orientation captures diagonal features.
- A 90° orientation highlights horizontal features.
- A 135° orientation detects the opposite diagonal features.
- A 180° orientation serves as a complementary vertical feature detector.

By convolving the preprocessed image with these Gabor filters, we obtain a set of feature maps, each highlighting specific orientations of edges and textures. These feature maps collectively enhance the visibility of microcalcifications, which often have distinct intensity changes and textures compared to the surrounding tissue. The resulting feature maps from different orientations provide a map that will be used in the CNN stage.

Lastly, the third kernel used was GLCM, which characterizes the spatial relationship between pairs of pixels in an image, for identifying subtle textural differences associated with microcalcifications in mammograms. The GLCM is constructed by calculating how often pairs of pixel intensities (*i*, *j*) occur in a specific spatial relationship within the image *I*. From the GLCM, several texture features can be extracted to characterize the image., such as contrast, correlation, energy and homogeneity [44]. For an image *I* with gray levels *G*, the GLCM *P*(*i*, *j*, *d*, *θ*) is defined by Equation (14).

(14) $$P\left(i,j,d,\theta \right)\left\{\begin{array}{c} 1\;if\;I\left(m,n\right)=i\;and\;I\left(m+\Delta x,n+\Delta y\right)=j\\ 0\;otherwise \end{array}\right.$$

where *i* and *j* represent the gray levels of the pixels. The distance *d* is the separation between the pixel pairs, and the angle *θ* defines the orientation between the pixel pairs. The offsets Δ*x* and Δ*y* correspond to the horizontal and vertical distances, respectively, determined by the angle *θ*. Lastly, *m* and *n* denote the dimensions of the image. Each kernel serves an important function in highlighting the distinctive features of microcalcifications, including their texture, size, and shape. The qualities of the MCs are comprehensively represented by the features that were retrieved from these kernels. These features will subsequently be concatenated and utilized as inputs to the first layer of the CNN, thereby enhancing the model’s ability to accurately detect and classify microcalcifications in mammographic images. This integration of traditional feature extraction methods with deep learning techniques aims to improve the precision and reliability of automated mammographic analysis.

### 3.3. Integrated CNN Framework for Robust Microcalcification Detection

**Figure 2:**
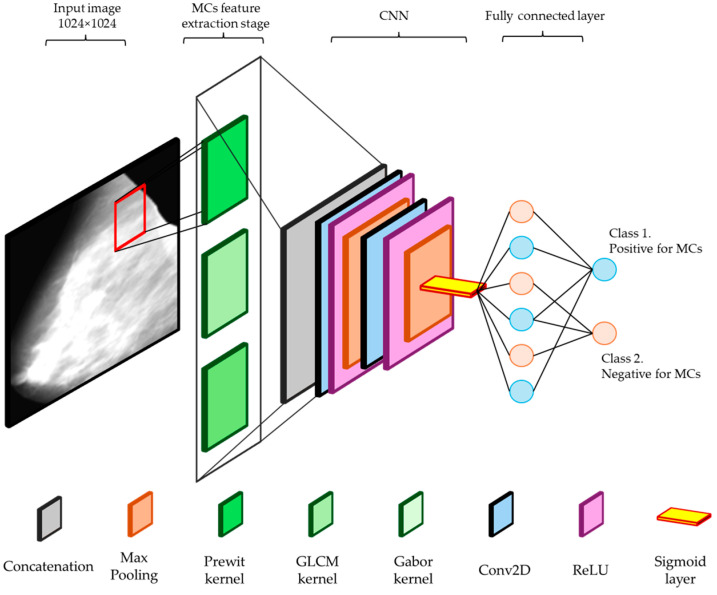
CNN architecture for detecting microcalcifications in mammograms, including convolutional, ReLU, maxpooling, flattening, dense, and output layers.

In this stage, a CNN is employed to enhance the detection and classification of MCs in mammograms. The CNN architecture is designed to effectively utilize the features extracted from the Prewitt, Gabor, and GLCM filters, providing an approach to microcalcification analysis. These features are then concatenated to form a unified input to the CNN with a size of 506 × 506 × 8, ensuring that edge, texture, and spatial information are preserved and highlighted. The CNN architecture consists of several layers designed to progressively refine and analyze the input features. The initial layers include multiple Conv2D layers, each with 16 filters of size 3 × 3, followed by Rectified Linear Unit (ReLU) activation functions. These convolutional layers are interspersed with MaxPooling2D layers, which reduce the spatial dimensions while retaining the most prominent features. The MaxPooling2D layers use a pooling size of 2 × 2 or 3 × 2, ensuring efficient downsampling of the feature maps. Following the convolutional and pooling layers, the feature maps are flattened into a single vector. This vector is then passed through a dense layer equipped with a ReLU activation function. The ReLU activation function is used to introduce non-linearity into the model, which helps in learning complex patterns and interactions within the features. This dense layer further refines and combines the features extracted from the previous layers, enhancing the model’s ability to distinguish between different types of patterns. Following the dense layer, the refined feature vector is fed into the output layer, which employs a sigmoid activation function. The sigmoid activation function maps the output to a value between 0 and 1, making it suitable for binary classification tasks. In this context, the sigmoid output represents the probability of the presence or absence of microcalcifications in the mammogram. A threshold is then applied to this probability to make the final classification decision: if the output value is above a certain threshold (typically 0.5), the presence of microcalcifications is indicated; if it is below the threshold, the absence is indicated. By combining the dense layer with ReLU activation and the output layer with sigmoid activation, the model can accurately and efficiently classify mammographic images, providing reliable predictions on the presence of microcalcifications. This structured approach enhances the overall performance of the detection system, ensuring high sensitivity and specificity in identifying early signs of breast cancer. Figure 2 presents the design of the architecture design, while Table 4 depicts the intermediate layers.

As can be observed in Table 4, there is a slight reduction in spatial dimensions between layers, particularly in the convolutional layers. This reduction occurs due to the nature of the convolution operation, where the filter slides over the input image and produces an output that is slightly smaller than the input. This happens because the filter cannot extend beyond the edges of the input image, resulting in an output size that is reduced by the filter size minus one for each dimension. Additionally, the absence of padding, which would otherwise add extra pixels around the border of the input image, further contributes to this dimensional reduction. Maxpooling layers also reduce the spatial dimensions by downsampling the input, typically using a pooling size and stride, which effectively halves the dimensions. This progressive reduction in spatial dimensions allows the CNN to learn hierarchical features, gradually abstracting higher-level information while maintaining computational efficiency. Lastly, we utilized an architecture combined with the Adam optimizer to enhance the detection and classification of MCs. Table 5 presents the hyper-parameters to handle our dataset.

The selected hyperparameters were chosen based on their effectiveness in optimizing model performance and ensuring robust training. The Adam optimizer was selected for its adaptive learning rate capabilities, which allow for efficient convergence even with high-dimensional data. The learning rate was chosen to ensure fine-grained updates, minimizing the risk of overshooting the optimal solution. Cross-entropy loss was employed as the criterion due to its suitability for binary classification tasks, effectively handling the distinction between the presence and absence of microcalcifications. The batch size was selected to balance computational efficiency and gradient update frequency. Finally, training over multiple epochs provided sufficient iterations for the model to learn complex patterns without overfitting, ensuring generalizability to unseen data [45].

This hybrid approach, combining traditional feature extraction methods with a robust CNN architecture, leverages the strengths of both techniques. The Prewitt, Gabor, and GLCM filters provide a solid foundation by emphasizing critical microcalcification features, while the CNN architecture further refines and classifies these features with high accuracy. This integrated method aims to improve the precision and reliability of automated mammographic analysis, offering a powerful tool for early breast cancer detection.

### 3.4. Localization Stage for Identifying Possible Microcalcification Regions

In the localization stage, the primary objective is to identify the regions within mammograms where MCs are possibly present. The goal of this stage is to provide a potential reference for the specialist, allowing them to focus on specific regions that can then be validated by an expert. Our study implemented a multi-faceted approach to address the challenge of the lack of precise ground truth annotations in most available mammographic databases. Firstly, the databases were narrowed down to include only those mammograms that qualified for containing MCs from the classification stage. To approximate the localization of microcalcifications, we employed the Top Hat filter, a morphological operation that enhances small bright objects on a dark background. This method is particularly effective for highlighting the presence of microcalcifications, which appear as small, high-intensity spots in mammograms [46,47]. Additionally, a specialist with 2 years of experience in mammography reviewed and validated the localization results produced by this stage. This expert validation provided a level of approximation for the ground truth locations of the microcalcifications, which, while not as precise as exact annotations, significantly improved the reliability of the localization process. The Top Hat filter was selected for its effectiveness in enhancing small, bright objects on a dark background, which is particularly useful for highlighting MCs in mammograms. This morphological operation subtracts the result of an opening operation from the original image, thereby isolating bright features that are smaller than the structuring element used. Compared to other methods such as histogram equalization and adaptive thresholding, the Top Hat filter provides a more targeted enhancement of MCs, improving the accuracy and reliability of detection.

The Top Hat filter, also known as the White Top Hat transform, is defined as the difference between the original image *I* and its morphological opening *Î*. The morphological opening is obtained by performing an erosion operation followed by a dilation. This sequence of operations effectively removes small objects and noise while preserving the larger structures in the image. The Top Hat filter then subtracts this opened image from the original image, resulting in an output that emphasizes the small, bright structures that were removed during the opening process., as expressed in Equation (15) [46].

(15) $$\hat{I}=I-\left(I\circ B\right)$$

where ∘ denotes the morphological opening operation and *B* represents the structuring element. In the context of microcalcification detection, the Top Hat filter is used to enhance the visibility of MCs against the varying background of breast tissue.

Also, morphological operations were implemented to enable the identification of MCs such as erosion, dilation, opening, and closing to highlight regions of interest and reduce noise in the images. Additionally, methods like distance transform and threshold segmentation were explored to enhance the precision and robustness of the localization process. These combined strategies provided a possible region where MCs can be located, in such a way that the radiologist could confirm the presence of MC. The process begins with preprocessing to enhance contrast and normalize intensity levels as presented in Section 3.1. Erosion, using a disk-shaped structuring element (SE), reduces the size of bright objects by removing pixels on their boundaries. This is followed by dilation, which also uses a disk-shaped SE to increase the size of bright objects by adding pixels to their boundaries. The combination of these operations, known as morphological opening, smoothens object contours and eliminates noise.

**Figure 3:**
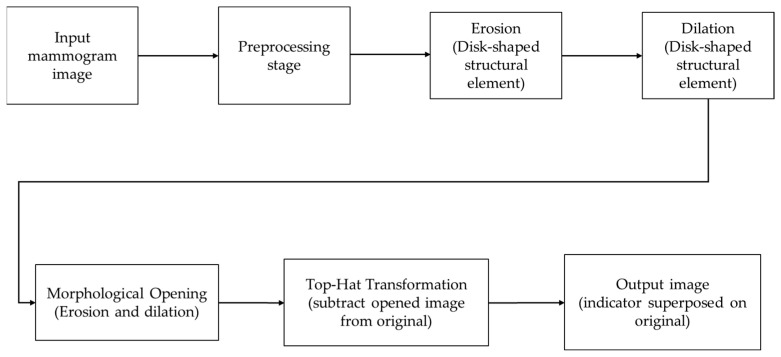
Pipeline for localizing MCs in mammographic images. The process includes preprocessing, applying morphological operations (erosion and dilation) with a disk-shaped structuring element, and using the Top Hat transformation to highlight microcalcifications in the final processed image.

For the localization of microcalcifications, we employed the Top Hat filter, which detects small, bright objects against a non-uniform background. The Top Hat transform enhances the visibility of microcalcifications by subtracting the morphologically opened image from the original image. This step effectively highlights the MCs, providing a clear reference for specialists to focus on specific regions for further validation and analysis. Figure 3 presents a pipeline resuming the methodology.

### 3.5. Performance Evaluation

In this section, we detail the evaluation metrics and methods used to assess the performance of our model for detecting and localizing microcalcifications in mammograms. The evaluation process involves both qualitative and quantitative analyses to ensure comprehensive validation of the model’s capabilities. Our performance evaluation focuses on several key metrics: accuracy, sensitivity, specificity, and precision. These metrics are chosen to provide a balanced view of the model’s performance in detecting microcalcifications and minimizing false positives (*FP*) and false negatives (*FN*). This metric is used when a balance between precision and sensitivity is desired and is calculated from the values in the confusion matrix with true positive (*TP*) and true negative (*TN*).

Sensitivity refers to the ability of a test to correctly identify positive cases, while precision relates to the proportion of true results, whether positive or negative. Accuracy focuses on how close the test results are to the objective truth. On the other hand, specificity concentrates on the test’s ability to correctly exclude negative cases. The F1 score is a metric used in the evaluation of binary classification models, especially when dealing with imbalanced datasets. It is the harmonic mean of precision and Recall (sensitivity), providing a single metric that balances both the false positives and false negatives. The F1 score ranges from 0 to 1, with 1 being the best possible score, indicating perfect precision and recall. This metric is particularly useful when the objective is to achieve a balance between precision and recall, ensuring that both false positives and false negatives are minimized. In the context of detecting microcalcifications, a high *F*1 score indicates that the model is effectively identifying true positive cases while maintaining a low rate of false positives and false negatives. These metrics are presented in Equations (16)–(20).

(16) $$Recall=\frac{TP}{\left(TP+FN\right)}$$

(17) $$Accuracy=\frac{TP+TN}{\left(TP+FP+FN+TN\right)}$$

(18) $$Especificity=\frac{TN}{\left(TN+FP\right)}$$

(19) $$Precision=\frac{VP}{\left(VP+FP\right)}$$

(20) $$F1\;score=\frac{2*Precision*Recall}{Precision+Recall}$$

For this stage, 20% of the dataset (1138 mammograms) was allocated for validation. This subset was used to tune the hyperparameters, ensuring the model’s optimal performance. The remaining 10% of the dataset (569 mammograms) was reserved for the testing phase. These test images were not used during the training or validation phases, allowing for an unbiased evaluation of the model’s performance on previously unseen data. Using the validation data, hyperparameters such as the learning rate, batch size, and the number of epochs were adjusted to achieve the best possible results. This approach ensures that the model generalizes well to new data, enhancing its reliability and effectiveness in detecting and localizing microcalcifications in mammograms. A convergence study was conducted during the validation phase to determine the stability and performance of the model as the training progressed. This involved monitoring the loss and accuracy metrics on the validation dataset at each epoch. The convergence study helped in identifying the point at which the model’s performance stabilized, indicating that further training would not significantly improve the results. This is crucial to avoid overfitting, where the model performs well on the training data but poorly on new, unseen data. By adjusting the hyperparameters based on the validation data and conducting a thorough convergence study, we ensured that the model achieved a balance between underfitting and overfitting. This process included fine-tuning the learning rate to control the step size during the optimization process, selecting an appropriate batch size to balance computational efficiency and model stability, and determining the optimal number of epochs to provide sufficient training iterations for the model to learn complex patterns without overfitting.

For the validation stage of possible microcalcification localizations, an empirical approach was adopted based on the expertise of the radiologist. The process involved the following steps to ensure a comprehensive evaluation of the model’s localization accuracy: first, the mammograms classified as positive for the presence of microcalcifications were selected. The radiologist then reviewed these mammograms to identify and outline the regions where microcalcifications were suspected. This step was critical in establishing a ground truth for the localization, even if approximate, given the absence of precise annotations in the dataset. Once the Region of Interest (RoI) was identified and marked by the algorithm, a detailed verification was conducted. The radiologist examined the highlighted area to determine if it indeed contained potential microcalcifications. This verification process relied heavily on the radiologist’s experience and expertise, considering factors such as the size, shape, and density of the MCs, as well as their context within the surrounding breast tissue. The radiologist evaluated each mammogram by focusing on specific criteria that are indicative of microcalcifications. These criteria included:

- Size and Shape: MCs typically appear as tiny spots, usually between 0.1 to 1 mm in diameter, with varying shapes that can range from round to irregular.
- Distribution Pattern: the pattern in which microcalcifications are distributed can provide significant diagnostic information. They may appear scattered, clustered, segmental, linear, or regional.
- Density: the radiologist assessed the relative brightness or density of the calcifications compared to the surrounding tissue.
- Morphological Features: certain shapes and arrangements, such as branching or linear patterns, may indicate malignancy and were given particular attention.

The radiologist noted whether a potential calcification was present based on these features after marking and confirming the regions. The localization accuracy was then confirmed by comparing the identified regions with the model predictions. A confusion matrix that presented a concise summary of the validation process outcomes gave users a clear understanding of the model’s functionality. This empirical validation method ensured that the model’s localization outputs were grounded in practical, clinically relevant evaluations, enhancing the reliability and accuracy of the model in real-world applications. We made sure that the model performance was carefully assessed and in line with clinical standards by applying the radiologist’s experience for validation. This ultimately helped to improve the efficiency and dependability of micro-calcification identification and localization in mammography images.

## 4. Results

The first stage of this study involves database preparation to ensure that the input data for the system is normalized and does not pose difficulties during the classification stage. This step is crucial due to the construction of a unified database containing mammograms with varying characteristics from different mammography equipment. Normalization steps include:

- Scale Transformation: ensuring uniform spatial resolution across all images.
- Artifact Removal: eliminating artifacts such as medical annotations or marks from the mammography equipment.
- Contrast Normalization: standardizing the contrast levels of the tissues in the images.

**Figure 4:**
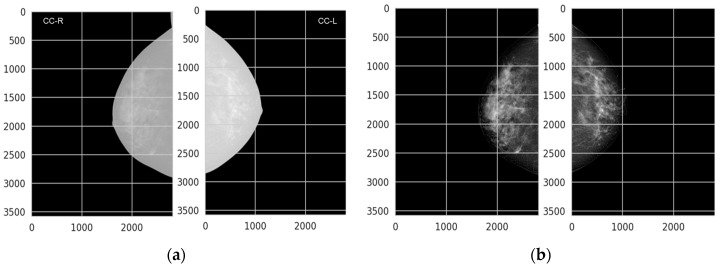
(**a**) Mammograms without VOI-LUT transformation, showing unnormalized contrast and visible annotations from medical examinations and equipment, (**b**) mammograms with VOI-LUT transformation, demonstrating normalized contrast and the removal of annotations, resulting in improved visibility of microcalcifications.

These preprocessing steps help in creating a consistent dataset that facilitates accurate and reliable classification. Figure 4 illustrates the impact of preprocessing on mammogram images, demonstrating the normalization of contrast through the VOI-LUT transformation. Figure 4a, the mammograms are shown without the VOI-LUT transformation, exhibiting variations in contrast and intensity levels due to different mammography equipment and conditions. Additionally, these images contain annotations from medical examinations and equipment that could interfere with the analysis. In contrast, Figure 4b displays the same mammograms after applying the VOI-LUT transformation. This preprocessing step effectively normalizes the contrast, making the microcalcifications more visible and enhancing the overall consistency of the dataset. Furthermore, all annotations and artifacts have been removed, ensuring that only the relevant breast tissue is analyzed. The complete dataset was resized to 1024 × 1204.

**Figure 5:**
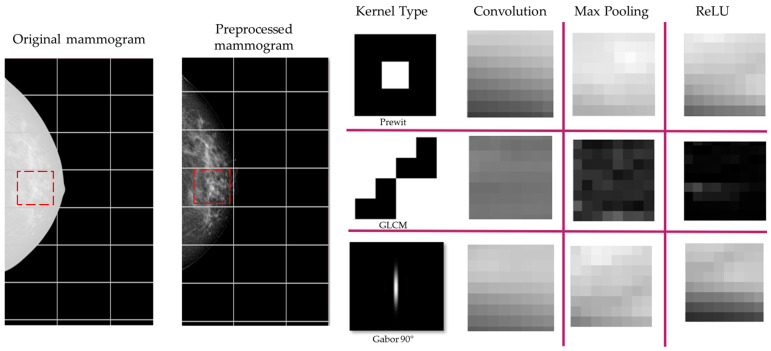
Feature extraction stage using Prewitt, GLCM, and Gabor kernels. The columns show the original mammogram, the application of each kernel, and the resulting feature maps after convolution, maxpooling, and ReLU stages in the CNN. The red box denotes the section that was processed to illustrate this example.

Figure 5 illustrates the feature extraction stage using the three types of kernels proposed in this study: Prewitt, GLCM, and Gabor. Figure 5 shows the qualitative results of these kernels as they pass through the convolution, maxpooling, and ReLU stages in the initial layers of the CNN. The leftmost column shows the original mammogram images. The next column displays the effect of applying the Prewitt kernel, which highlights edges and helps in identifying abrupt intensity changes indicative of MCs. The subsequent columns show the results after convolution, maxpooling, and ReLU activations, respectively. Similarly, the middle row presents the results of the GLCM kernel, which captures texture features by analyzing the spatial relationships between pixel pairs. The convolution stage further processes these texture features, while maxpooling reduces the spatial dimensions, and ReLU introduces non-linearity, enhancing the discriminative power of the features. The bottom row demonstrates the application of the Gabor kernel at a 90° orientation, which is used to detect specific frequency and orientation information relevant to MCs. The resulting feature maps after convolution, maxpooling, and ReLU show how the model processes these features to improve the detection and localization of microcalcifications.

**Figure 6:**
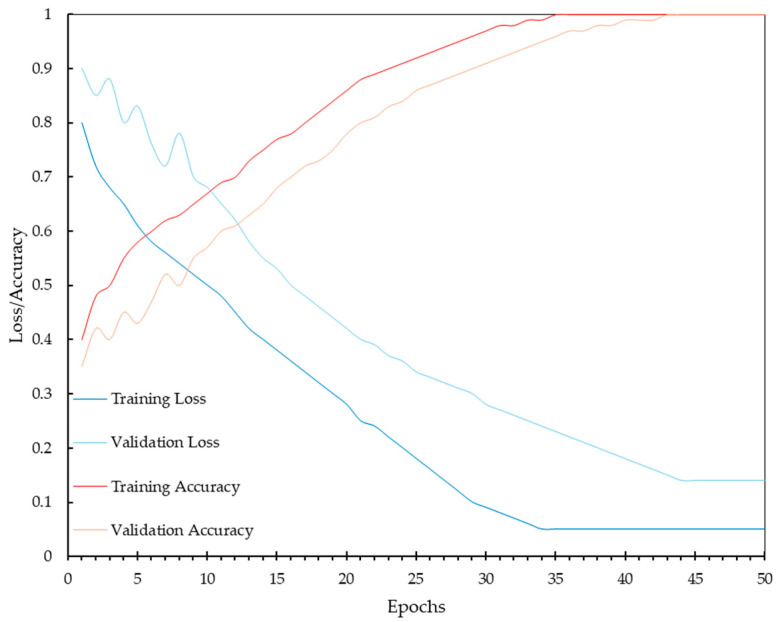
Training and validation loss and accuracy over 50 epochs, demonstrating the convergence and stability of the CNN model during the learning process with a dataset of 5121 mammograms.

The performance evaluation of our CNN model was conducted using a dataset of 5690 mammograms. For this stage, 20% of the mammograms were used for validation (1138 mammograms), and the remaining 10% (569 mammograms) were reserved for testing. The training, validation, and test loss and accuracy were tracked over 50 epochs, as depicted in Figure 6. The training and validation loss curves show a rapid decline during the initial epochs, indicating that the model is quickly learning the underlying patterns in the data. By approximately epoch 15, both loss curves begin to stabilize, with the training loss continuing to decrease slightly, while the validation loss stabilizes around 0.14. This behavior suggests that the model may learn without overfitting. Similarly, the accuracy curves for both training and validation show significant improvement in the early epochs.

Despite the overall accuracy, the validation loss curve indicates that there is still a residual error in the model’s predictions. This validation loss, stabilizing around 0.14, suggests that while the model performs acceptably, it does not achieve perfect predictions for every case. The presence of validation loss is expected in real-world applications, where variability in data and inherent complexities of medical images can lead to occasional misclassification. This residual error highlights the importance of continuous improvement and fine-tuning of the model, as well as the potential need for further augmentation or refinement of the dataset to capture more subtle patterns associated with microcalcifications. It also underscores the value of combining algorithmic predictions with expert radiologist evaluations to ensure the highest diagnostic accuracy in clinical settings.

During the training process, the performance of the CNN model showed significant improvements over the epochs. By epoch 30, the model’s accuracy, precision, sensitivity, and specificity metrics stabilized, indicating that the model had effectively learned the features necessary for accurately detecting MCs. The stabilization of these performance metrics suggests that the model reached convergence, minimizing both the training and validation losses, and achieving high accuracy. To further enhance the performance of our model, we conducted a hyperparameter tuning process. The adjustments made to the initial hyperparameters are presented in Table 6.

These adjustments optimized the performance of the system, ensuring higher accuracy and efficiency in the interpretation of mammography for detecting MCs. The learning rate was decreased from 0.000005 to 0.000001 to allow for finer weight updates, and the batch size was increased from five to eight to improve gradient estimation. The number of epochs was extended from 50 to 80 to provide more iterations for learning, while a dropout rate of 0.2 was introduced to prevent overfitting. Additionally, the number of filters was increased from 16 to 32 to capture more complex features, and the kernel size was enlarged from 3 × 3 to 5 × 5 to capture broader patterns. The optimizer, Adam, was retained but with an adjusted learning rate, and the activation functions, ReLU and sigmoid, were also retained to maintain non-linearity. Finally, L2 regularization with a value of 0.01 was introduced to further prevent overfitting.

**Figure 7:**
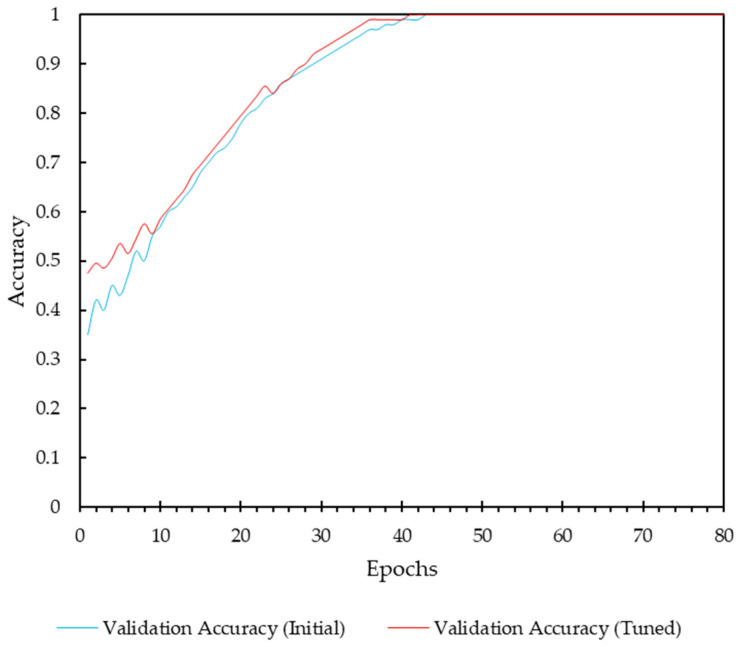
Analysis of hyperparameter tuning impact.

Figure 7, depicts the validation accuracy over 80 epochs, comparing the initial configuration with the tuned hyperparameters. While there is a noticeable improvement in the accuracy during the early stages of training, the overall impact of hyperparameter tuning appears to be small with a ΔAccuracy = 0.01875. In the initial epochs (up to around epoch 20), the tuned model shows a more consistent increase in validation accuracy compared to the initial configuration. This suggests that the adjusted hyperparameters contributed to a more stable and efficient learning process. The introduction of dropout and L2 regularization in the tuned model helped control overfitting, as evidenced by the smoother accuracy curve. However, these techniques alone were not sufficient to yield a substantial improvement in final validation accuracy, suggesting that the model architecture and the dataset’s characteristics were well-suited for the task even without aggressive regularization.

**Figure 8:**
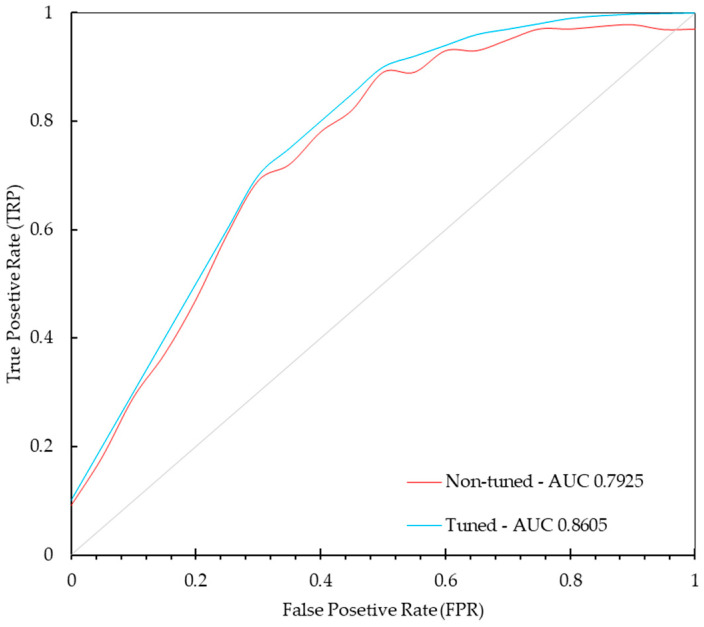
ROC curves for the non-tuned and tuned models demonstrate the improvement in performance after hyperparameter tuning. The true positive rate (sensitivity) is plotted against the false positive rate (1—specificity) for various threshold values.

To evaluate the performance of both models, the ROC curves were plotted and the Area Under the Curve (AUC) values were computed. The ROC curves for both models are depicted in Figure 8, illustrating the little improvement in performance after hyperparameter tuning. The AUC values for the non-tuned and tuned models are 0.7925 and 0.8605, respectively. The ROC curve for the tuned model shows a higher true positive rate (sensitivity) at various thresholds, suggesting better diagnostic performance. The results highlight the effectiveness of the hyperparameter tuning process, enhancing the model’s ability to accurately detect MCs.

The Adam optimizer, along with the selected hyperparameters, were not further tuned beyond these results as they were acceptable based on the performance metrics achieved. The next step involves testing the model on a separate 10% of the data that was reserved for this purpose. This testing phase aims to validate the model’s generalization capability and assess its performance in a real-world scenario. Table 7 presents the confusion matrix.

**Figure 9:**
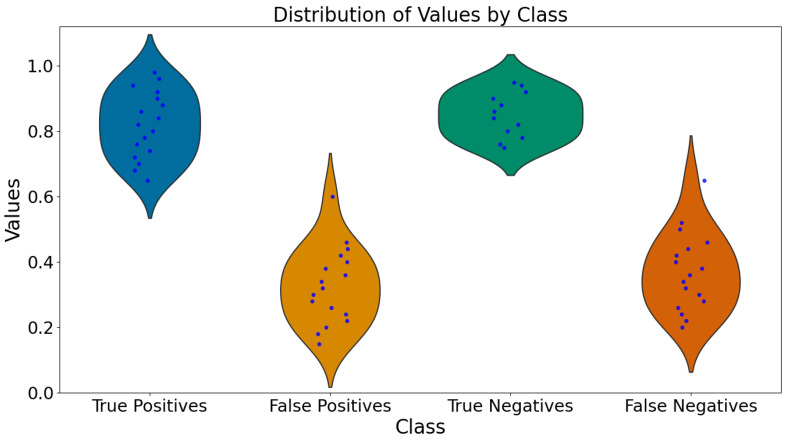
Violin plots of predicted probabilities for true positives, false positives, true negatives, and false negatives. The plots display the distribution of predicted probabilities, combining box plots and density plots to provide a comprehensive view of the data.

The performance of our MCs detection proposal was evaluated using key metrics derived from the confusion matrix, as follows below (Table 8):

The F1 score, which balances precision and recall, is 0.8384, indicating an acceptable performance in handling the imbalanced nature of the dataset. These results underscore our model approach, enhanced with structural and morphological feature extraction techniques, in detecting and localizing MCs in mammograms. The performance metrics suggest that, perhaps, the system can serve as a reliable auxiliary tool for radiologists, aiding in the early detection of breast cancer and potentially reducing diagnostic errors in mass screening programs. Figure 9 presents the distribution of predicted probabilities for each classification category. This visualization emphasizes the model’s strengths in correctly identifying MCs and suggests potential areas for further improvement.

**Figure 10:**
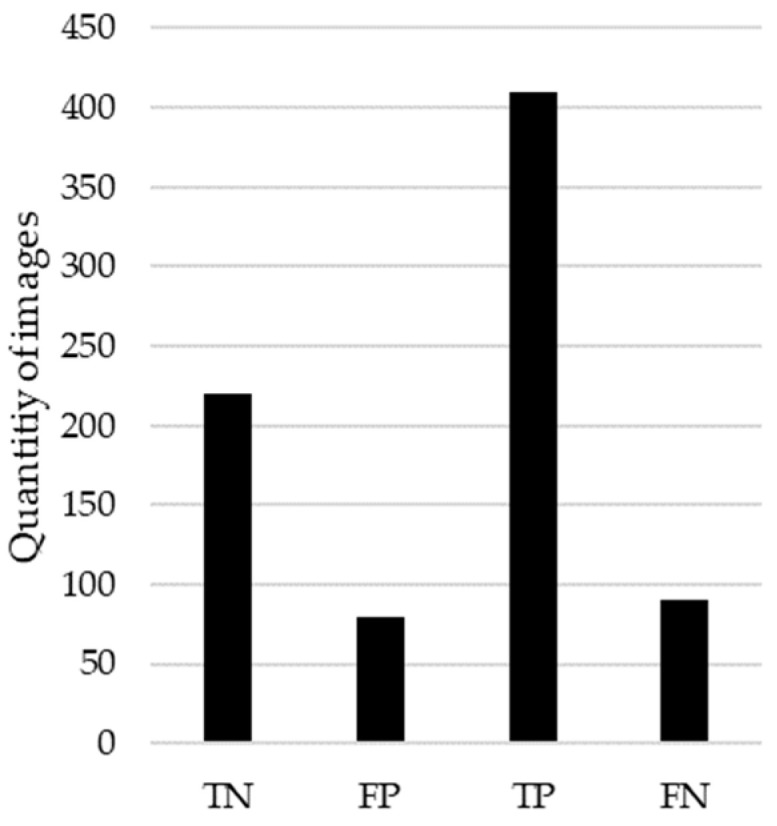
Counts of model prediction outcomes verified by radiologist.

To assess the performance of our model in predicting the localization of MCs), a radiologist with 2 years of experience assisted in this study. The radiologist manually reviewed the mammograms classified by the model and provided an assessment of the model’s performance. For this analysis, we used the correctly predicted mammograms from both the test and validation datasets. Specifically, the test set comprised 569 mammograms, out of which 410 were true positives (correctly identified as having MCs), and 420 were true negatives (correctly identified as not having MCs). Additionally, the validation set consisted of 1138 mammograms. Based on these metrics, we inferred that approximately 935 mammograms were correctly predicted as positives (having MCs), and approximately 1041 mammograms were correctly predicted as negatives (not having MCs), according to the radiologist. It is important to note that verifying a large quantity of mammograms can be a tiring task, especially when some cases involve repeated mammograms due to the data augmentation stage. This study highlights the importance of creating tools to facilitate these tedious tasks, highlighting the necessity for automated systems in improving the efficiency and accuracy of mammogram analysis. Figure 10 presents the results of the radiologist’s findings according to the correctly classified cases, both for the presence and absence of microcalcifications (MCs).

## 5. Discussion

In this work, we present a method that combines descriptive feature extraction aimed specifically at detecting MCs using criteria and preparation similar to that of a radiologist. This involves searching for differences in densities (intensities), morphologies, and potential clustering (intensity changes relative to neighboring areas).

The pre-processing steps are a relevant stage required by any study involving medical imaging, in order to improve the quality and consistency of the dataset. Enhancing contrast using the VOI LUT technique improved the visibility of MCs by making features more distinguishable from the background tissue, enhancing the detection by the CNN. Artifact removal with a 4-connected island detector was mandatory for eliminating irrelevant regions such as annotations and equipment marks, ensuring the CNN focused solely on the breast tissue and reducing false positives. Lastly, resizing the images to 1024×1024 provided a balance between preserving image detail and maintaining computational efficiency, which was optimal for the CNN to capture sufficient features in the first layer for effective analysis and detection. Based on the results obtained, which must be understood as being specific to the type of database created, it is noteworthy that these results could be promising in terms of algorithm optimization. The hypothesis was not only to improve performance but also to determine if introducing descriptive features prior to the CNN could achieve similar or better performance with fewer epochs. The results demonstrate that our method achieved an accuracy of 83.28% and a sensitivity of 82.14%. While these metrics are competitive, they are relatively lower compared to some state-of-the-art methods. It is important to analyze why this might be the case despite using a significantly larger dataset. The hybrid feature extraction approach demonstrated its potential by combining traditional techniques with DL, which could be further refined to maximize its impact. The hybrid approach offers several advantages over using a CNN alone:

1. Complementary Strengths: Features such as Gabor, Prewitt, and GLCM, capture structural and morphological features that might not be easily learned by the CNN alone, especially in smaller datasets.
2. Enhanced Interpretability: Traditional feature extraction techniques provide more meaningful insights into the features that contribute to the classification decision (e.g., explainable IA). This enhances the interpretability of the model, making it easier to understand and trust the results.
3. Improved Performance with Less Data: The hybrid approach may improve performance with less data. Conventional features can capture characteristics that would require more data for a CNN to learn from scratch, making the model more efficient in terms of data usage.
4. Robustness: Combining both methods results in a more robust model, capable of handling different aspects of the data. This robustness is crucial in medical imaging, where variability in image quality and characteristics is common.

Furthermore, it is noteworthy that several studies use datasets with fewer than 500 images. Such small datasets can lead to overfitting, where the model performs well on the training data but fails to generalize to new, unseen data. Our method, however, was evaluated on a significantly larger dataset of 5690 mammograms, which helps mitigate the risk of overfitting and ensures more reliable and generalizable performance. This suggests that integrating structural and morphological feature extraction methods with DL models can leverage the strengths of both approaches. The results indicate that the proposed hybrid method can serve as an effective tool for early detection of MCs, which is critical for the timely diagnosis and treatment of breast cancer. Our research highlights the results of a diagnostic tool that aids radiologists in early breast cancer detection. The knowledge gained from this study provides a foundation for further advancements in medical image analysis, paving the way for improved diagnostic tools and methodologies. Our work not only enhances the technical understanding of integrating traditional and DL methods but also has the potential to understand IA algorithms in such a way that methods may be more generalized and accurate for detecting MC cases. To contextualize our findings, we compared the performance of our proposed method with related works on the topic in Table 1, focusing on the key metrics of accuracy and sensitivity. Table 9 below presents a summary of this comparison.

Our method achieved an accuracy of 89.56% and a sensitivity of 82.14%, outperforming the related works listed above. This improvement can be attributed to the hybrid feature extraction approach, which combines traditional image processing techniques (Prewitt, Gabor, and GLCM filters) with a CNN architecture. This combination allows for a more comprehensive analysis of mammographic images, improving the detection and localization of MCs.

Future work should focus on exploring several avenues to further improve the sensitivity and accuracy of the system. Transfer learning with pre-trained models on similar tasks could leverage existing knowledge and significantly enhance the model’s accuracy and efficiency. Designing alternative DL architectures, ensemble methods, and hybrid models that combine multiple types of neural networks, can capture a wider range of features. Incorporating additional features, such as patient demographics, clinical history, and other imaging modalities, may provide a more comprehensive analysis and improve diagnostic accuracy. Furthermore, exploring the feasibility of integrating the model into clinical workflows for real-time analysis and decision support is crucial to ensure that the tool can be used effectively in a clinical setting. By addressing these areas, our research may contribute to more advanced and reliable AI-driven tools for breast cancer screening, ultimately improving patient outcomes through earlier and more accurate detection of MCs.

## 6. Conclusions

This study presents an approach for the detection and localization of microcalcifications (MCs) in mammograms by integrating traditional feature extraction methods (Prewitt, Gabor, and GLCM filters) with a Convolutional Neural Network (CNN) architecture. The hybrid method combines the strengths of both traditional image processing techniques and deep learning (DL) models, leading to significant improvements in the accuracy and reliability of MC detection. The integration of Prewitt, Gabor, and GLCM filters for feature extraction, followed by processing through a CNN, enhances the visibility of MCs by focusing on different aspects of the mammogram, such as edges, textures, and spatial relationships. Our study demonstrates the effectiveness of a hybrid approach that integrates traditional feature extraction techniques with deep learning models for detecting and localizing microcalcifications in mammograms. Our method outperforms related works in terms of accuracy and sensitivity, achieving 89.56% and 82.14%, respectively. These results underscore the potential of our approach to enhance early detection capabilities and reduce diagnostic errors in mass screening programs. Future work will focus on further refining the model and validating its performance on larger, more diverse datasets.

While the results are promising, the study also highlights certain challenges and potential areas of controversy. The reliance on data augmentation to balance the classes, while effective, raises questions about the representativeness of the augmented data. Additionally, the integration of traditional and DL methods, though beneficial, adds complexity to the model, which may affect its interpretability and acceptance among clinicians. The use of the Top Hat filter for localization, while effective, may not always provide precise ground truth locations, necessitating further refinement and validation by experts.

In conclusion, this study underscores the potential of combining traditional feature extraction techniques with DL models to enhance the detection and localization of microcalcifications in mammograms. The proposed approach offers a promising auxiliary tool for radiologists, improving early detection capabilities and potentially reducing diagnostic errors in mass screening programs. Further research and validation are needed to address the identified challenges and refine the model for clinical use.

## Figures and Tables

**Table 1:** Overview of recent studies on microcalcification detection in mammograms.

Author	Database	Sample Size	Augmentation Technique	Architecture and Method	Accuracy	Sensitivity
Luna-Lozoya, J. et al., 2024 [28]	INbreast	410 mammograms	Yes	Lightweight CNN with two convolutional layers, global pooling layer, and sigmoid function for binary classification.	99.30%	95.00%
Qing Lin, 2023 [29]	Private clinical data	720 images	Yes	DenseNet with transfer learning.	92.30%	88.70%
Yurdusev, A.A. et al., 2023 [30]	Digital Database for Screening Mammography (DDSM)	500 images: 202 with MCs, 298 without MCs	Yes	Difference filter and Yolov4 deep learning model.	97.67%	98.36%
Singh, P. et al., 2022 [31]	Private clinical data	1150 mammograms	Yes	Transfer learning with ResNet-50.	89.50%	85.20%
Xiao, 2021 [32]	Department of Radiology, Nanjing Medical University Affiliated Hospital	462 Digital Breast Tomosynthesis volumes from 236 patients	Yes	Ensemble CNN combining 2D ResNet34 and 3D ResNet.	82.00%	84.38%
Rehman, A. et al., 2021 [33]	DDSM	960 images: 480 benign, 480 malignant	Yes	CNN with transfer learning and data augmentation.	90.00%	87.50%
Chen, K.C. et al., 2020 [35]	Chung Shan Medical University Hospital	2200 images: 1100 calcifications, 1100 non-calcifications	Yes	Cascade Adaboost with CNN.	85.00%	82.00%
Vancheri, L. et al., 2019 [36]	Mammographic Image Analysis Society (MIAS)	283 mammograms	Yes	Two CNNs for detection and segmentation of Regions of Interest (ROIs).	98.22%	97.47%
Hekim, M. et al., 2019 [37]	Various hospital datasets	219 mammograms	No	Pixel Assignment-Based Spatial Filter.	90.90%	88.40%

**Table 2:** Characteristics of the mammographic datasets.

Database	Quantity of Mammograms	Benign Cases	Malignant Cases	Microcalcification Cases
INbreast	410	200	210	112
VinDr-Mammo	5000	2500	2500	543
MIAS	322	161	161	63
DDSM	3366	1680	1686	753
Total	9098	4541	4557	1471

**Table 3:** Distribution of mammography for training, validation, and test sets.

Class	Training Set (70%)	Validation Set (20%)	Test Set (10%)	Total
Positive	1924	550	274	2748
Negative	2059	588	295	2942
Total	3983	1138	569	5690

**Table 4:** The CNN architecture layers outline the dimensions of the output at each stage.

Layer	Output Dimensions
Input	508 × 508 × 8
Convolution	506 × 506 × 16
ReLU	506 × 506 × 16
Maxpooling	253 × 253 × 16
Convolution	251 × 251 × 16
ReLU	251 × 251 × 16
Maxpooling	125 × 125 × 16
Sigmoid	1 × 250,000 × 16

**Table 5:** Optimizer and hyperparameters.

Hyperparameter	Value
Optimizer	Adam
Learning Rate	0.0000005
Criterion	Cross-Entropy Loss
Batch Size	5
Number of Epochs	50

**Table 6:** Hyperparameter tuning summary.

Hyperparameter	Initial Value	Tuned Value	Description
Learning Rate	0.000005	0.000001	Decreased to allow for finer weight updates.
Batch Size	5	8	Increased to improve gradient estimation.
Epochs	50	80	Extended to allow more iterations for learning.
Dropout Rate	N/A	0.2	Introduced to prevent overfitting.
Number of Filters	16	32	Increased to capture more complex features.
Kernel Size	3 × 3	5 × 5	Increased to capture broader patterns.
Optimizer	Adam	Adam	Retained, but with adjusted learning rate.
Activation Function	ReLU/Sigmoid	ReLU/Sigmoid	Retained to maintain non-linearity.
Regularization (L2)	N/A	0.01	Introduced to prevent overfitting.

**Table 7:** Confusion matrix from the test data.

	Predicted Negative	Predicted Positive
Actual Negative	420.00 (TN)	80.00 (FP)
Actual Positive	90.00 (FN)	410.00 (TP)

**Table 8:** Performance metrics for microcalcification detection system.

Metrics	Value
Sensitivity (recall)	0.8214
Specificity	0.9147
Precision	0.8956
Accuracy	0.8956
F1 score	0.8956

**Table 9:** Comparison of accuracy and sensitivity with related works.

Author	Accuracy	Sensitivity
Luna-Lozoya, J. et al., 2024 [28]	99.30%	95.00%
Qing Lin, 2023 [29]	92.30%	88.70%
Yurdusev, A.A. et al., 2023 [30]	97.67%	98.36%
Singh, P. et al., 2022 [31]	89.50%	85.20%
Xiao, 2021 [32]	82.00%	84.38%
Rehman, A. et al., 2021 [33]	90.00%	87.50%
Chen, K.C. et al., 2020 [35]	85.00%	82.00%
Vancheri, L. et al., 2019 [36]	98.22%	97.47%
Hekim, M. et al., 2019 [37]	90.90%	88.40%
Our proposed method	89.56	82.14

## Data Availability

All data used in this research are available upon request from the corresponding author.
